# Correlation between motor behavior and age‐related intervertebral disc degeneration in cynomolgus monkeys

**DOI:** 10.1002/jsp2.1183

**Published:** 2022-01-01

**Authors:** Jianmin Wang, Peixuan Zhu, Ximin Pan, Jun Yang, Shijun Wang, Wentao Wang, Baoliang Li, Zhengya Zhu, Tao Tang, Dafu Chen, Manman Gao, Zhiyu Zhou

**Affiliations:** ^1^ Department of Orthopedic Surgery The Seventh Affiliated Hospital of Sun Yat‐sen University Shenzhen China; ^2^ International Medical Center Foresea Life Insurance Guangzhou General Hospital Guangzhou China; ^3^ Department of Radiology The Sixth Affiliated Hospital(Gastrointestinal Hospital), Sun Yat‐sen University Guangzhou China; ^4^ Department of Radiology Longkou Second People's Hospital Yantai China; ^5^ Department of the Joint and Bone Surgery Yantaishan Hospital Yantai China; ^6^ Laboratory of Bone Tissue Engineering, Beijing Laboratory of Biomedical Materials Beijing Research Institute of Orthopedics and Traumatology, Beijing JiShuiTan Hospital Beijing China; ^7^ Department of Sport Medicine Inst Translat Med, The First Affiliated Hospital of Shenzhen University, Shenzhen Second People's Hospital Shenzhen China; ^8^ Guangdong Provincial Key Laboratory of Orthopedics and Traumatology The First Affiliated Hospital of Sun Yat‐sen University Guangzhou China; ^9^ Shenzhen Key Laboratory of Anti‐aging and Regenerative Medicine, Department of Medical Cell Biology and Genetics Health Sciences Center, Shenzhen University Shenzhen China

**Keywords:** ageing, cynomolgus monkey, intervertebral disc degeneration, motor behavior, pain

## Abstract

**Background:**

The motor behavior in patients with lumbar intervertebral disc degeneration (IDD) and animal models should be changed due to pain. However, there does not seem to be a strong correlation between IDD and motor behavior. Therefore, it is necessary to understand the correlation between motor behavior and age‐related IDD.

**Methods:**

Twenty‐one healthy male cynomolgus monkeys (*Macaca fascicularis*) distributed across the age range were included in this study. The experimental animals were divided into two groups: caged group (n = 14) and free‐range group (n = 7). The data of IDD and motor behavior were obtained through magnetic resonance imaging (MRI) and PrimateScan Automatic Behavior Analysis System. More than 20 basic motor behaviors could be recorded and quantified, and then reclassified into 9 combined categories. We defined the sum of the duration of activity‐related combined categories as the total duration of activity in 3 hours. The activity zone of the cynomolgus monkeys in the cage could be divided into top and bottom zones. Analyze the correlation between motor behavior and IDD.

**Results:**

Age was correlated with both Pfirrmann grades (*r* = .700; *P* < .001) and T2 values (*r* = −.369; *P* < .001). The T2 value in the caged group was 45.97 ± 8.35 ms, which was significantly lower than the 55.90 ± 8.73 ms in the free‐range group (*P* < .001). The mean T2 values were positively correlated with hanging duration (*r* = .548, *P* < .05), the total duration of activity (*r* = .496, *P* < .05), and top zone duration (*r* = .541, *P* < .05).

**Conclusions:**

There is an interactional relationship between IDD and motor behavior. Motor behavior could be used as one of the diagnostic indicators of IDD. It could also be used to infer the presence or extent of IDD in animal models. Avoiding a sedentary lifestyle and engaging in exercise in daily life could alleviate IDD.

## Graphical abstract

There is an interactional relationship between intervertebral disc degeneration (IDD) and motor behavior. Motor behavior could be used as one of the diagnostic indicators of IDD. It could also be used to infer the presence or extent of IDD in animal models. Avoiding a sedentary lifestyle and engaging in exercise in daily life could alleviate IDD.

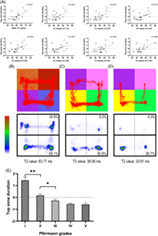

## 1 INTRODUCTION

At a certain stage of life, approximately 70% to 85% of people suffer from low back pain.^1^ In the United States, 14% (13 million) of patients seeking medical help and treatment have low back pain as their chief complaint.^2^ According to statistics, approximately 637 million people in the world are affected by low back pain,^3^ which seriously affects the quality of life of patients and increases the economic burden on families and society.^4^, ^5^ It is generally believed that lumbar intervertebral disc degeneration (IDD) is one of the main causes of low back pain.^6^, ^7^, ^8^, ^9^ IDD is an extremely common disease that occurs with ageing.^8^, ^10^, ^11^, ^12^, ^13^

Motor behavior includes all movements from unconscious twitches to goal‐oriented actions, and its development spans the entire life cycle from the first fetal movement to the last breathe.^14^ Common motor behaviors are standing, walking, running, jumping, sitting, lying, bending, waist rotation, and so forth. Theoretically, the motor behavior in patients with IDD should be changed due to pain,^15^ however, there does not seem to be a strong correlation between the degree of IDD and the presence and severity of symptoms.^16^, ^17^ Clinical symptoms often do not match the degree of IDD. Therefore, it is necessary to understand the characteristics of IDD‐related motor behavior, which is helpful for the clinical diagnosis of IDD.

However, motor behavior in humans is affected in many ways. In addition to severity of disease, it also changes with factors such as gender, age, and psychological variables. For example, positive magnetic resonance imaging (MRI) results will increase the stress of patients, and patients will change their motor behavior due to fear.^18^, ^19^, ^20^, ^21^ On the other hand, it is difficult to separate the influencing factors on motor behavior from lifestyle, environment, and other factors. Therefore, a more reliable method to study the relationship between IDD and motor behavior is through an animal model of IDD.^22^

A variety of techniques have been used to develop experimental animal models of IDD. Norcross et al^22^ injected chondroitinase ABC into the proximal two intervertebral discs (IVDs) in the tails of rats and found IDD in the second week after the operation. Holm et al.^23^ created an animal model similar to human disc degeneration by damaging the endplate. In domestic pigs, the L3‐4 disc was exposed through a left retroperitoneal approach. A 3.5‐mm drill bit was inserted from the middle of the L4 vertebral body. At an angle of 45° relative to the center of the endplate, a hole was drilled in the NP. L3‐4 disc degeneration occurred after 3 months. Although these animal models provide approaches for the study of IDD, they are artificially induced IDD models and could not fully simulate the IDD resulting from the process of human ageing.^24^ There are a small number of naturally occurring animal models, however most of which involve sand rats or multiple canine breeds,^25^, ^26^, ^27^, ^28^ the anatomical structure of the spine and motor behavior characteristics are quite different from those of humans, which makes it difficult to generalize these findings to humans.

Non‐human primates are very similar to humans in terms of spinal structure and motor behavior characteristics. Age‐related spontaneous IDD models could simulate the process of human lumbar disc degeneration well. There are few studies on spontaneous animal models of IDD in non‐human primates. Because animals could not describe pain, changes in experimental animals' motor behavior usually were used to infer the presence or extent of pain.^24^ Therefore, understanding the animal behavioral characteristics is helpful for intensive research on IDD.

This research aimed to study the relationship between motor behavior and lumbar IDD. We observed the spontaneous degeneration process of IVDs and the characteristics of motor behavior in non‐human primates through MRI examinations and the PrimateScan Automatic Behavior Analysis System.

## 2 METHODS

### 2.1 Experimental animals

The study protocol was reviewed and approved by the institutional review board and ethics committee of Institute of Zoology, Guangdong Academy of Sciences (No. G2Z20210103). The animal living conditions complied with the national standards of primate laboratories. Experimental animals were purchased from Guangzhou Topgene Biotechnology Inc., with detailed birth and research records and quarantine certificates. The raising and nursing of animals and the motor behavior analysis were all completed at the Institute of Zoology, Guangdong Academy of Sciences, and the MRI was implemented at Foresea Life Insurance Guangzhou General Hospital. An experienced veterinarian was in charge of animal care. Motor behavior testing and MRI were all carried out by specialized technicians.

Twenty‐one male cynomolgus monkeys (*Macaca fascicularis*), with a mean age of 12.19 ± 4.00 years (range, 7‐20 years), which can be converted to equivalent human age by using a 1:3.5 ratio,^29^ and a mean body weight of 8.25 ± 1.89 kg (range, 5‐12 kg) were used in this study.

Cynomolgus monkeys between 7 and 9 years of age were categorized as “young” (n = 7), and those between 12 and 14 years of age were categorized as “middle‐aged” (n = 8). Six cynomolgus monkeys that were 15 years of age or older were categorized as “elderly.”

The experimental animals were divided into two groups: caged group (n = 14) and free‐range group (n = 7). Caged housing means that the animals were raised in cages for 2‐3 years, with limited space for activities. The size of the cage was 90*80*85 cm. Free‐range housing means that the animals were raised in a natural environment, and the animals had a large space for activities. Experimental animals had lived in a free‐range environment from birth. To avoid the influence of the experimental environment on the results, free‐range animals were raised in a caged environment for at least 3 days before the start of the experiment.

### 2.2 MRI acquisition

MRI examination was performed under anesthesia. The cynomolgus monkeys were fasted for 8 hours and then were anesthetized with tiletamine hydrochloride and zolazepam hydrochloride (Zoletil 50, 8‐10 mg/kg, 50 mg/mL; Virbac, France) by intramuscular injection. Aspiration of vomiting was avoided by tracheal intubation. The subjects were kept warm during the MRI examination.

All MRI were performed with a 3.0‐Tesla magnetic resonance scanner (General Electric Company). The posture of the experimental animals was supine. The MRI protocol for the lumbar spine included sagittal T2‐weighted imaging (T2WI) sequences and biochemical sequences for sagittal T2 mapping. The imaging parameters of MRI are listed in Table 1.

**TABLE 1:** Parameters for MRI of cynomolgus monkeys

Parameter	T2WI	T2 mapping
Pulse sequence	FSE	FSE
Repetition time (ms)	2000	1200
Echo time (ms)	102	TE*
Field of view (mm)	20 × 20	20 × 20
Pixel bandwidth (Hz)	325.5	651
Voxel size (mm)	0.8 × 0.8	1.0 × 1.0
Slice thickness (mm)	3	3
Interslice gap (mm)	0.6	0.6
Number of slices	7	7
Turbo factor	16	…
Acquisition time	1 min 40s	4 min 12 s

*Note*: TE*: 7.4, 14.8, 22.3, 29.7, 37.1, 44.5, 52, 59.4.

### 2.3 T2 mapping quantification

**FIGURE 1:**
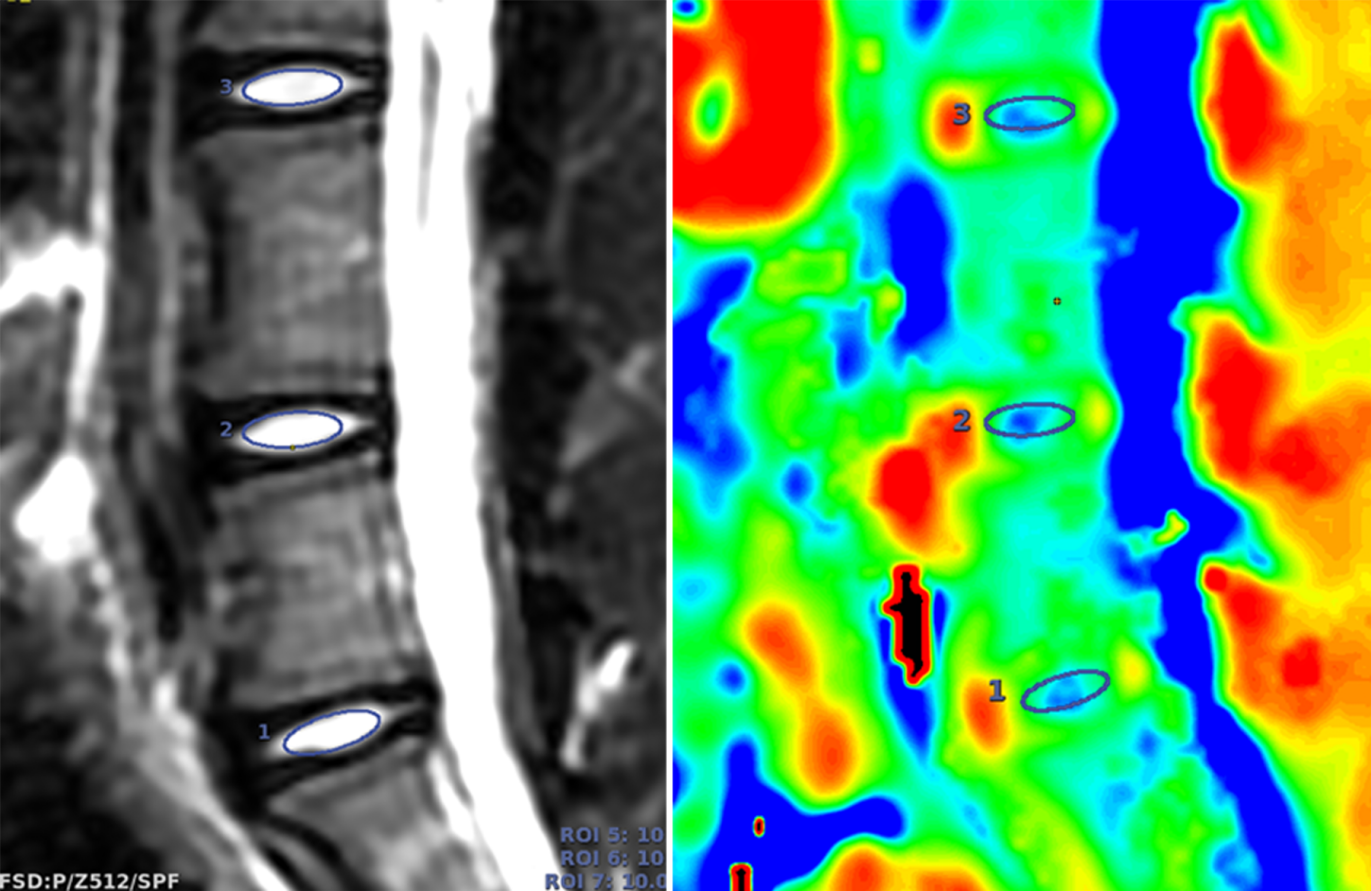
Measuring T2 values of the ROI of the NP. The ROI was an ellipse with an area of 10 mm^2^ located in the center of the NP. The long axis of the ellipse was parallel to the anterior and posterior sagittal lines of the IVD, and the upper and lower sides of the ellipse could not touch the cartilage endplate. The ROI was copied to the corresponding T2 mapping pseudo‐color map. IVD, intervertebral disc; ROI, region of interest

T2 mapping image analyses were conducted by using AW VolumeShare 7 (General Electric Company, United States). The region of interest (ROI) was manually outlined on the mid‐sagittal T2WI and then the T2 value was measured and recorded (Figure 1). To reduce the variance, the measurement was repeated 3 times at different time points (with an interval of more than 1 week), and the average value was obtained as the T2 value of the target IVD.

### 2.4 Pfirrmann grading

Three professional physicians, with 8 to 26 years of clinical work experience, graded all lumbar IVDs on the T2WI according to the classification scale described by Pfirrmann et al.^30^ The above process was performed under the same display and the same brightness mode. All the MR images were analyzed three times by the physicians on separate occasions, with a minimum interval of 1 week between imaging reviews. Regarding disc grading that showed differences, the discrepancies were resolved by the three physicians after consultation.

### 2.5 Motor behavior

The experimental animals moved freely in a cage with a size of 110*120*85 cm and were continuously videotaped by a camera for 3 hours. All animals had free access to food and water. All video acquisitions occurred in the daytime. The PrimateScan Automatic Behavior Analysis System (PrimateScan, HRT, CleverSys Inc., USA), a video‐based analysis system of spontaneous behaviors, was used to analyze the animal's motor behavior. For each cynomolgus monkey, MRI and Motor behavior were not distinguished in order. However, the acquisition of MRI and Motor behavior data was completed within two month.

More than 20 basic motor behaviors could be recorded and quantified by PrimateScan Automatic Behavior Analysis System. Based on previous literature reports^31^, ^32^ and the movement characteristics of cynomolgus monkeys, these basic motor behaviors were reclassified into nine combined categories (hanging, horizontal locomotion, vertical locomotion, climb, stand up, come down, sleep, stationary, and sitting). The combined motor behavior categories of hanging, horizontal locomotion, vertical locomotion, climb and stand up are related to activity. We defined the sum of the duration of these five combined motor behavior categories as the total duration of activity in 3 hours. The PrimateScan Automatic Behavior Analysis System also tracked the monkeys' activity in the top and bottom areas of the testing chamber and recorded the duration spent in each zone. Combined motor behavior categories, the total duration of activity and zone duration were used for statistical analysis.

### 2.6 Statistical analyses

Statistical analyses were performed using IBM SPSS Statistics for Windows v.26 (SPSS, Chicago). Differences between the groups were evaluated by one‐way analysis of variance (ANOVA) or *t* test. Pearson's and Spearman's correlation analyses were used to analyze the correlation between IDD and age; Pfirrmann grades and T2 values; and T2 values and indicators of motor behavior. Multivariate linear regression analysis was used to describe the influencing factors on T2 values and motor behavior. For all analyses, a *P* < .05 was considered significant.

## 3 RESULTS

### 3.1 Characteristics of IDD

#### 3.1.1 Correlation between IDD and age

**FIGURE 2:**
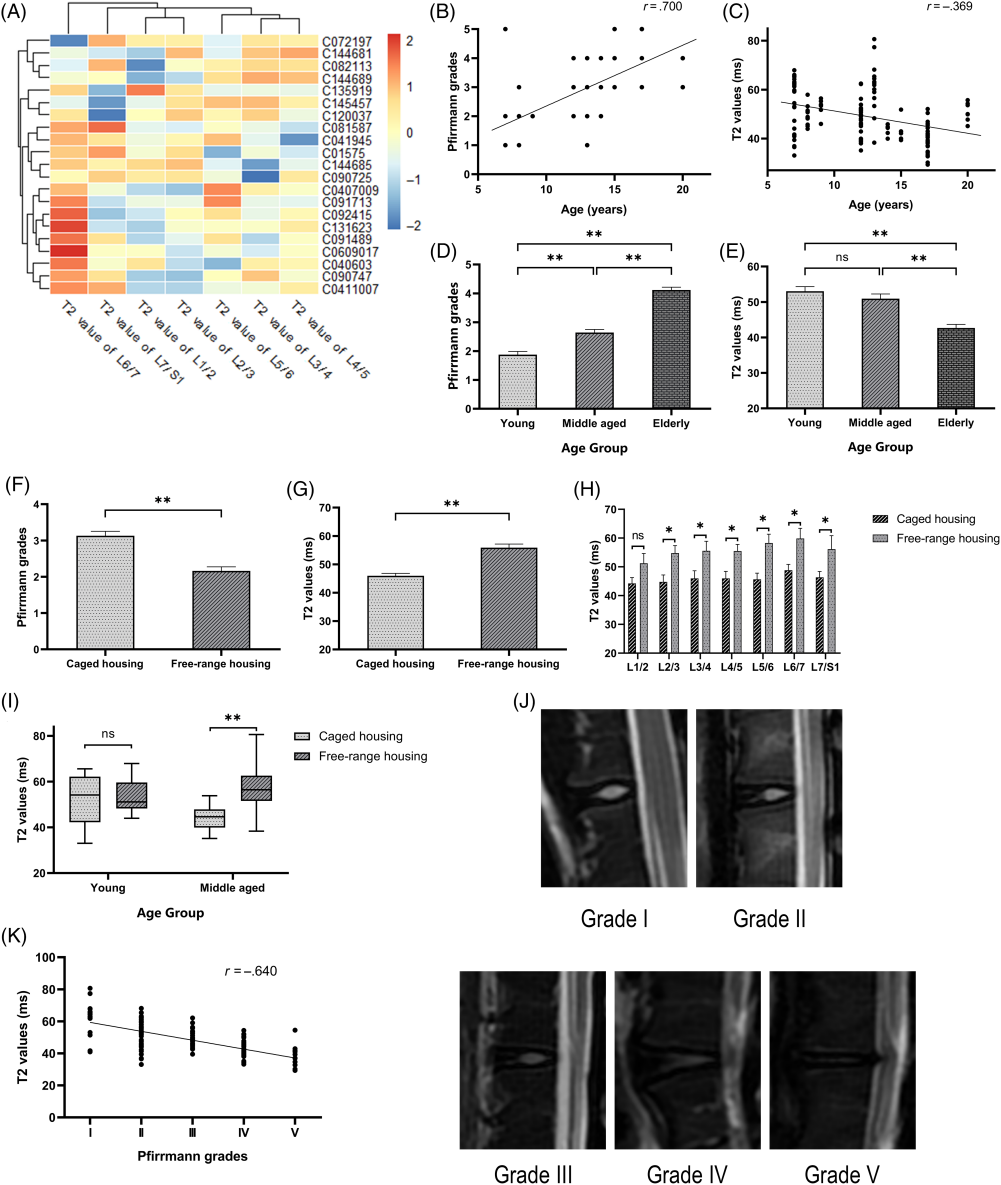
Characteristics of intervertebral disc degeneration (IDD). (A) Heatmap showing the T2 values of each lumbar segment of each cynomolgus monkey. (B,C) Scatter plots show that age was correlated with both Pfirrmann grades (*r* = .700; *P* < .001) and T2 values (*r* = −.369; *P* < .001). Pfirrmann grades (D) and T2 values (E) of cynomolgus monkey lumbar intervertebral discs (IVDs) in the different age groups. Pfirrmann grades (F) and T2 values (G) of the cynomolgus monkey lumbar IVDs in different living conditions. (H) Effects of living conditions on the IDD in each segment. (I) The box plot graph (median and interquartile range) shows influence of living conditions on T2 values across the different age groups. (J) T2WI of lumbar IVDs related to Pfirrmann grades in cynomolgus monkeys. (K) Pfirrmann grades were significantly negatively correlated with T2 values (*P* < .001). * *P* < .05; ** *P* < .001; ns, not significant; mean ± SEM; n = 147 (49 young, 56 middle‐aged, and 42 elderly)

The lumbar discs of all cynomolgus monkeys were scanned by MRI, and T2WI and T2 mapping sequences of 147 IVDs (49 young, 56 middle‐aged, and 42 elderly) were obtained (Figure 2A). Age was correlated with both Pfirrmann grades (*r* = .700, *P* < .001) and T2 values (*r* = −.369, *P* < .001; Figure 2B,C). With increasing age, Pfirrmann grades gradually increased (*P* < .001), and T2 values gradually decreased (*P* < .001 between the middle‐aged group and the elderly group, *P* > .05 between the young group and the middle‐aged group; Figure 2D,E).

#### 3.1.2 Impact of living conditions on IDD

Differences in the age and weight between the two groups were not statistically significant (*P* > .05). The numbers of IVDs obtained from the caged group and the free‐range group were 98 (66.67%) and 49 (33.33%), respectively. The Pfirrmann grade for the caged group was significantly greater than the free‐range group (*P* < .001; Figure 2F). The T2 value in the caged group was significantly lower than the free‐range group (*P* < .001; Figure 2G). The T2 value in each segment of the free‐range group was greater than that of the caged group (*P* > .05 in L1/2 and *P* < .05 in all other segments; Figure 2H).

In the young group (Figure 2I), the T2 values of the IVDs in cages and the free‐range animals were 52.52 ± 10.06 ms and 53.72 ± 7.66 ms, respectively, and the difference was not statistically significant (*P* = .649). In the middle‐aged group (Figure 2I), the T2 values of the IVDs in the caged and free‐range experimental animals were 44.37 ± 4.49 ms and 57.53 ± 9.25 ms, respectively, and this difference was statistically significant (*P* < .001). In the elderly group, there were no free‐range animals.

#### 3.1.3 Correlation analysis between Pfirrmann grades and T2 values

A total of 147 IVDs in cynomolgus monkeys were scanned. These discs were divided into five grades according to the Pfirrmann grading standards (Figure 2J). The number and percentage of IVDs at each grade were as follows: grade I, 19 (12.93%); grade II, 46 (31.29%); grade III, 38 (25.85%); grade IV, 32 (21.77%); and grade V, 12 (8.16%). As the Pfirrmann grade level increased, the T2 value gradually decreased. There was no significant difference in the T2 value between Pfirrmann grade IV and grade V (*P* = .056), but the difference in the T2 values for each other pair of grades was significant (*P* < .05). Spearman correlation analysis demonstrated a negative correlation between Pfirrmann grades and T2 values (*r* = −.640, *P* < .001; Figure 2K).

### 3.2 Characteristics of motor behaviors

#### 3.2.1 Combined motor behavior categories

**FIGURE 3:**
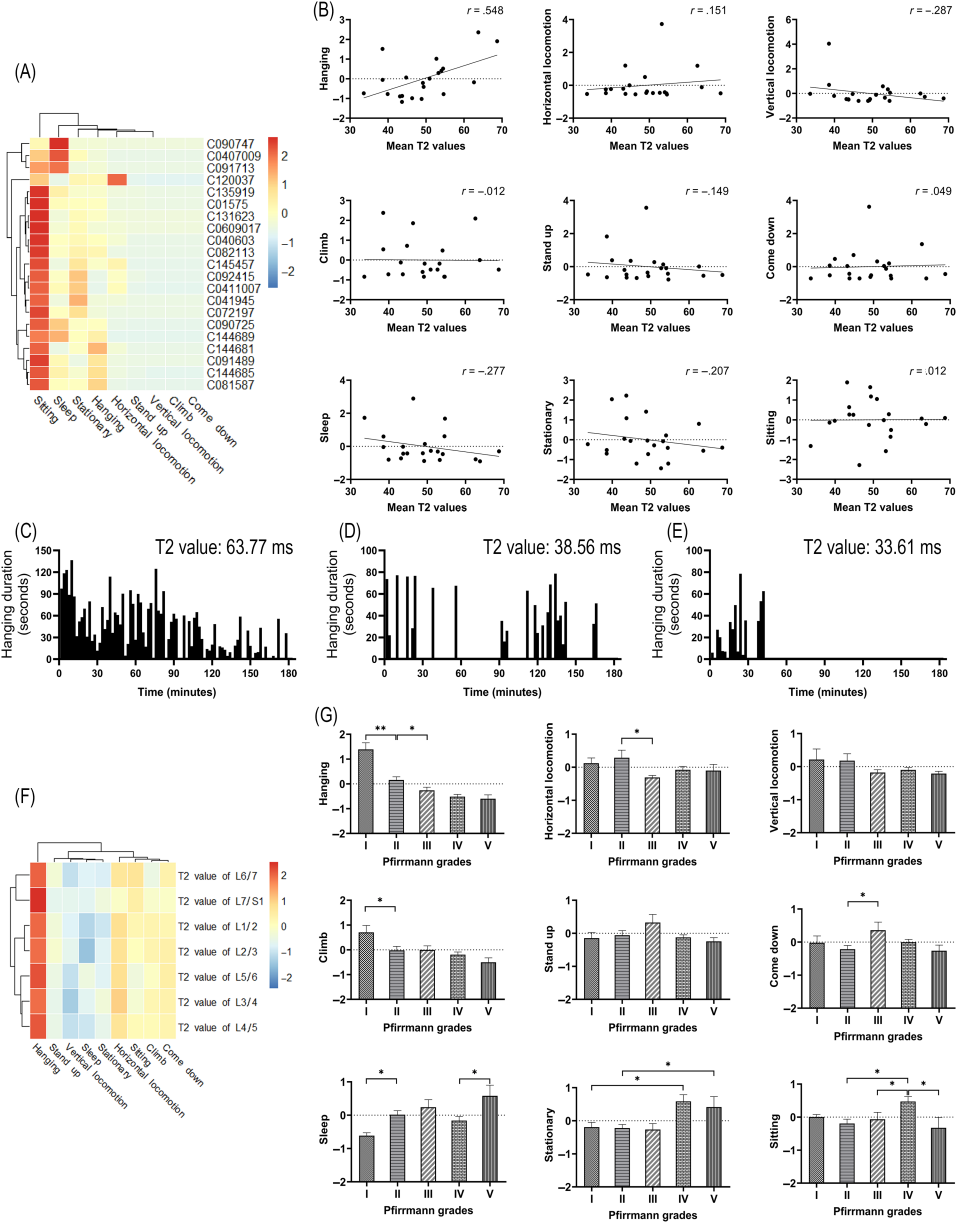
Characteristics of combined motor behavior categories. (A) Heatmap showing the combined motor behavior categories for each cynomolgus monkey. (B) Mean T2 values in each cynomolgus monkey and combined motor behavior categories (*P* = .01 in hanging and *P* > .05 in the other eight combined categories). (C‐E) Examples presentations of the hanging durations of experimental animals with different T2 values. The T2 values and hanging duration of these three cynomolgus monkeys were (C) 63.77 ms and 3606.81 s, (D) 38.56 ms and 1149.36 s, and (E) 33.61 ms and 455.49 s, respectively. (F) Heat map of the correlation coefficients between the T2 values of each segment and the combined categories of motor behavior. (G) Durations for each combined category across different Pfirrmann grades. Values on the vertical axes in (B) and (G) have been standardize. * *P* < .05; ** *P* < .001; mean ± SEM; n = 147 in (A), (F), (G); n = 21 in (B)

Twenty‐seven basic motor behaviors and nine combined motor behavior categories for each cynomolgus monkey were obtained (Figure 3A). The mean T2 values were positively correlated with hanging (*r* = .548, *P* = .010), but the correlations between mean T2 values and the other eight combined motor behavior categories were not statistically significant (*P* > .05; Figure 3B). Figure 3C‐E illustrates the hanging duration of three experimental animals with different mean T2 values. With the mean T2 value increased, the hanging duration gradually decreased.

The T2 values of all IVD segments were positively correlated with hanging and were statistically significant (*P* < .05) and the T2 values of all IVD segments had no statistically significant correlations with the remaining eight combined motor behavior categories (*P* > .05; Figure 3F). As the Pfirrmann grades increased, the hanging duration gradually decreased (Figure 3G). There was a great decrease in hanging duration from grade I (2590.72 ± 1155.10 ms) to grade III (943.40 ± 771.14 ms; *P* < .05). This pattern of change was not found in other eight combined motor behavior categories (Figure 3G).

#### 3.2.2 Total duration of activity

**FIGURE 4:**
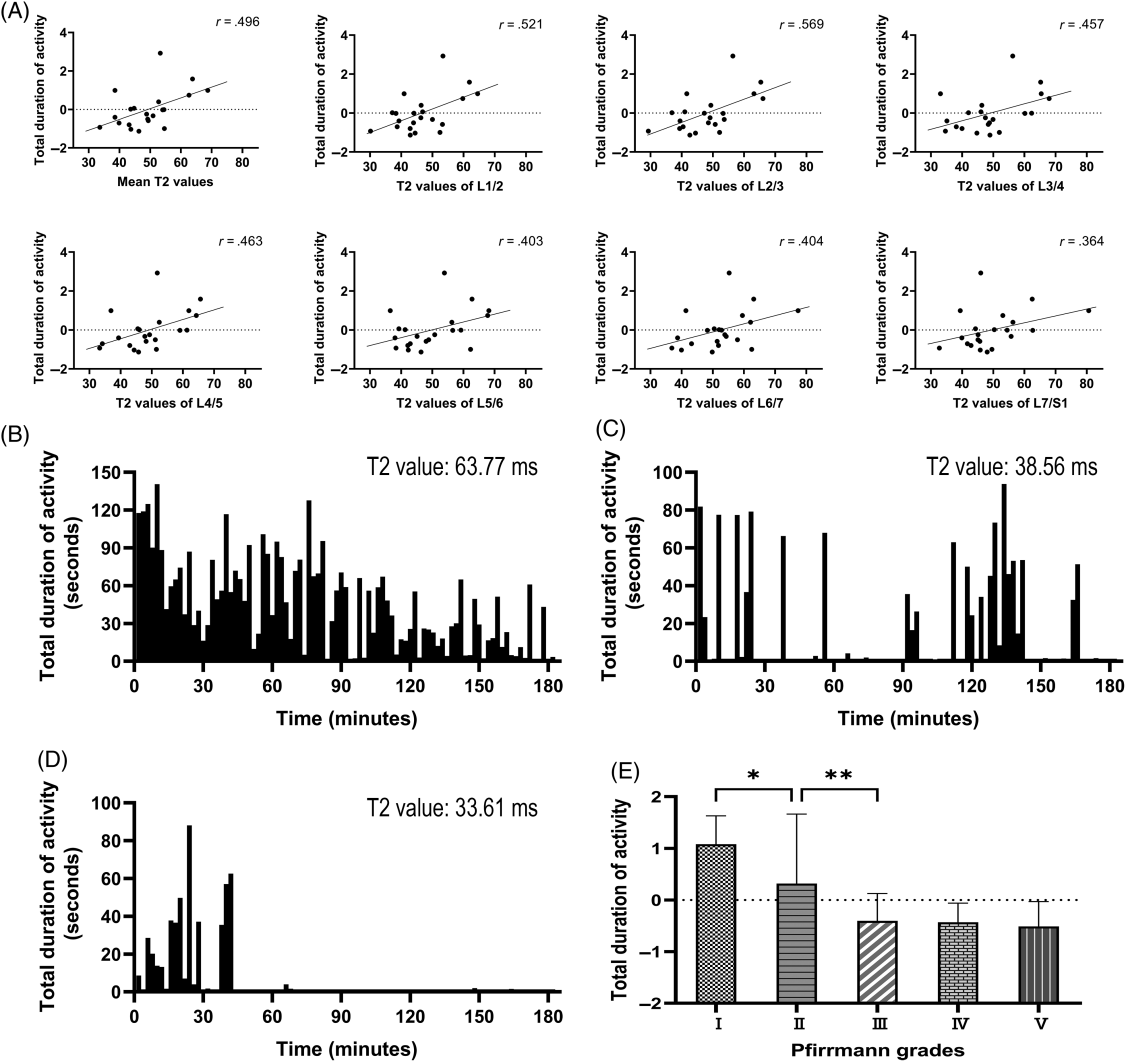
Characteristics of total duration of activity. (A) T2 values and the total duration of activity (*P* < .05 in mean T2 values and T2 values of L1/2, L2/3, L3/4, and L4/5; *P* > .05 in T2 values of L5/6, L6/7, L7/S1). (B‐D) Examples presentation of the total duration of activity of experimental animals with different T2 values. The mean T2 values and the total duration of activity of these three cynomolgus monkeys were (B) 63.77 ms and 4086.12 s, (C) 38.56 ms and 1260.09 s, and (D) 33.61 ms and 514.57 s, respectively. (E) Total duration of activity across Pfirrmann grades. Values on the vertical axes in (A) and (E) have been standardize. * *P* < .05; ** *P* < .001; mean ± SEM; n = 21 in (A), n = 147 in (E)

Total duration of activity was positively correlated with the mean T2 values and T2 values of all segments. The correlations in mean T2 values and T2 values of L1/2, L2/3, L3/4 and L4/5 were statistically significant (*P* < .05), however the correlations between the T2 values of the remaining segments and the total duration of activity were not statistically significant (*P* > .05; Figure 4A). Through the changes of the total duration of activity in three experimental animals with different mean T2 values, this trend can be seen intuitively (Figure 4B‐D). The comparison of the total duration of activity in different Pfirrmann grades also showed this trend (Figure 4E). With the Pfirrmann grade increased, the total duration of activity gradually decreased.

#### 3.2.3 Zone duration

**FIGURE 5:**
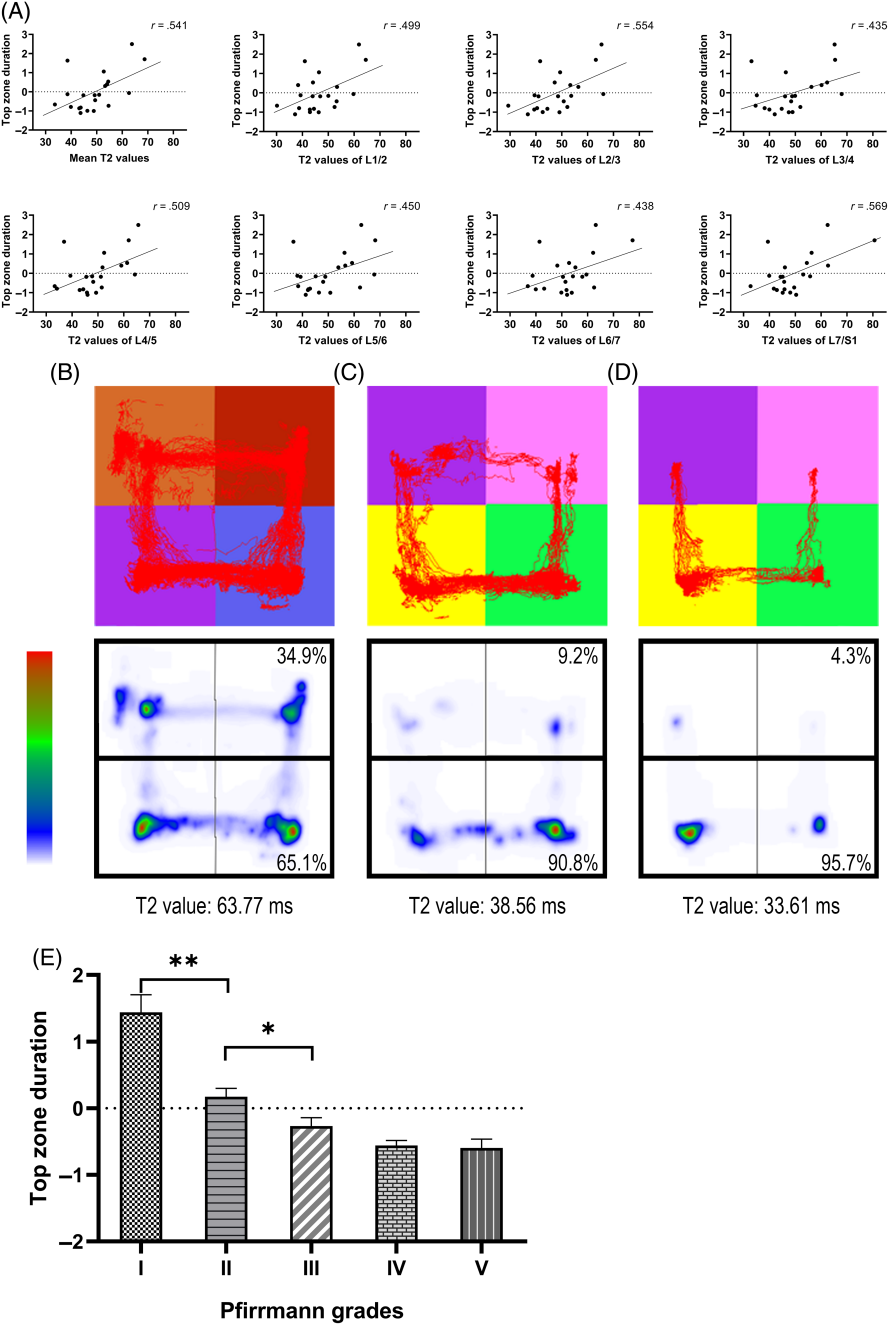
Characteristics of zone duration. (A) T2 values and top zone duration (*P* < .05). n = 21. (B‐D) Examples presentation of activity trajectories and zone durations for experimental animals with different T2 values. As the T2 value decreases, the percentage of top zone duration gradually decreases. (E) Top zone duration across Pfirrmann grades. Values on the vertical axes in (A) and (E) have been standardize. * *P* < .05; ** *P* < .001; mean ± SEM; n = 147

Top zone duration was positively correlated with the mean T2 values and T2 values of all segments (*P* < .05; Figure 5A). Examples presentation of activity trajectories and zone durations for experimental animals with different T2 values clearly demonstrate this change pattern (Figure 5B‐D). The comparison of top zone duration in different Pfirrmann grades also showed that with the Pfirrmann grade increased, the top zone duration gradually decreased (Figure 5E).

### 3.3 Influencing factors on T2 values and motor behavior

**FIGURE 6:**
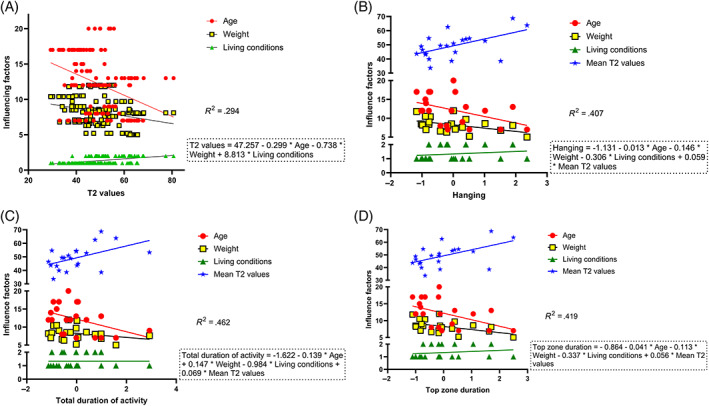
Multiple linear regression analysis of T2 values and motor behavior. (A) The main influencing factor on T2 values was living conditions. (B‐D) The mean T2 values were the most important factor influencing the motor behavior. Duration of motor behavior have been standardized; n = 147 in (A); n = 21 in (B‐D)

The T2 values of cynomolgus monkeys were negatively correlated with age, weight, and living conditions (*P* < .001). Multiple linear analyses showed a significant correlation between the T2 values and living conditions (*P* < .05), but not between the T2 values and weight, age (*P* > .05; Figure 6A). In multiple linear regression analysis, the mean T2 values was significantly correlated with hanging duration (*R* ^2^ = .407, *β* = .520, *P* < .05; Figure 6B) and total duration of activity (*R* ^2^ = .462, *β* = .613, *P* < .05; Figure 6C). The mean T2 values was the main influencing factor of top zone duration (*R* ^2^ = .419, *β* = .494); however, it was not statistically significant (*P* = .056; Figure 6D).

## 4 DISCUSSION

Motor behavior usually changes in patients with IDD, but the clinical symptoms do not seem to reflect the degree of IDD.^31^, ^32^ The relationship between IDD and motor behavior remains a mystery.^33^, ^34^ PrimateScan Automatic Behavior Analysis System was used to analyze the characteristics of IDD‐related motor behavior in cynomolgus monkeys in this experiment. The results suggested that motor behavior, such as hanging duration, total duration of activity, and top zone duration, was markedly correlated with IDD.

IDD could cause changes in motor behavior. Katie Mitchell et al^21^ reported that patients with low back pain have less movement of the lower lumbar spine on the sagittal plane and more movement of the lower extremity. The study of Shengzheng Kuai et al found that patients with lumbar disc herniation mainly restrict the motion of lower lumbar and upper lumbar in the spinal region during the five activities of daily living and pelvic rotation is an important method to compensate for the limited lumbar motion.^15^ However, the prior studies on IDD‐related motor behavior was limited to the range of motion of related local joints. The total duration of activity in the age‐related spontaneous IDD model of cynomolgus monkeys was analyzed. The results suggested that in the IDD model, as the degree of IDD worsened, the total duration of activity of the cynomolgus monkeys gradually decreased. The results of this study are in line with previous reports. Compared with the previous studies, this study observes motor behavior as a whole rather than the local joints of the subjects.

Ageing could also lead to a decrease in the total duration of activity,^35^ and the experimental data suggested that age was negatively correlated with the total duration of activity. However, multiple linear regression analysis suggested that T2 value rather than age was the main influencing factor of the total duration of activity. The T2 values were positively correlated with the top zone duration and the mean T2 values was the main influencing factor on the top zone duration. The activity of the animals was tracked in the top and bottom zones of the testing chamber. The activities of the cynomolgus monkeys in the top zone required actions such as climbing, hanging, or jumping. When animals suffer from IDD, the above actions will cause pain, and the animals will reduce their engagement in these actions to avoid pain.

Motor behavior and IDD could influence each other. There are many possible causes of IDD, such as occupational factors, obesity, smoking, genetics, age, and trauma.^36^, ^37^ A study conducted in a Japanese population^38^ showed that IDD in young adult men was related to overweight and abdominal obesity. Another study^39^ found that smokers have a higher risk of cervical IDD than non‐smokers. Matsui et al^40^ found that IDD has a strong familial predisposition. Motor behavior is a common cause that can contribute to IDD. In a cross‐sectional prevalence study, Evans et al^41^ found that the incidence of IDD was higher in sedentary women, but IDD was not found in women who were frequently active. Jensen et al^37^ found that compared with the general working population in Denmark, professional drivers were more prone to degenerative changes in the lumbar spine. The main risk factor that affected IDD in the cynomolgus monkeys was the living condition. Animals in caged group spent more time in states of bending and sitting. This is similar to human living habits such as long‐term deskwork coupled with a lack of exercise. Animals in free‐range group could freely perform various actions, such as running, jumping, and waist rotation. This is most similar to human life habits such as physical exercise. This research found that the IVDs of the animals in the caged group underwent more severe degeneration than the free‐range group. The results of the current research and related research reports indicate that living habits are influencing factors on IDD and long‐term deskwork and lack of exercise may aggravate IDD.

For free‐range monkeys, they are always fighting. Old and weak monkeys cannot protect themselves, and they are often injured by young and strong monkeys. Therefore, when monkeys are older or in poor health, they will be separated from the group of monkeys and then raised in cages. Therefore, there were no elderly animals in the free‐range group included in this experiment. To exclude the influence of age groups on the experimental results, we further analyzed the middle‐aged and young groups. In the middle‐aged group, caged housing could aggravate the IDD, but in the young group, the living condition did not yet have an impact on IDD. These results indicate that the effect of living condition on IDD is more likely to occur in the middle‐aged group.

In humans, the visual analogue scale is the most commonly used method of assessing pain, but this method could not be used in animal models.^37^ Motor behavior is the most commonly used method to assess the degree of pain in animal models.^42^ Zhang et al^31^ analyzed 38 basic behaviors of mice and reclassified these behaviors into eight combined categories. They found that hanging behavior was impaired in the pain model. The impairments in hanging behavior were dependent on the intensity of the pain stimulus and could be reversed by analgesics. Twenty‐seven basic motor behaviors of cynomolgus monkeys were reclassified into nine combined motor behavior categories. We found that the degree of IDD was correlated with hanging in the age‐related IDD model of cynomolgus monkeys, and the correlations with the other 8 combination categories were not statistically significant. Hanging was defined as any movement of the animal at the top of the cage. Multiple linear regression analysis was performed to predict the association of age, weight, and living conditions and the mean T2 values on hanging duration. Of the variables tested, the mean T2 values independently predicted hanging duration. These findings provide a new perspective for our understanding of non‐human primate IDD models.

We used MRI to assess the characteristics of IDD in cynomolgus monkeys. The study found that with increasing age, the IVDs in cynomolgus monkeys underwent degeneration. These data indicated that the IDD of the cynomolgus monkeys was age‐related spontaneous degeneration, which was consistent with previous reports in the literature.^12^, ^13^

In the process of transition from scientific theory to clinical application, the study of animal models is crucial.^25^ As an upright walking animal, human beings have unique characteristics of motor behavior and the biomechanics of intervertebral discs. At the same time, it is not completely clear whether artificial intervention induced degeneration of the intervertebral disc could completely simulate the pathological process of human IDD. Compared with other species animal models of IDD and animal models induced by other mechanisms, the age‐related IDD model of cynomolgus monkeys could better simulate the pathological process of human IDD. At the same time, the influence of occupational factors, smoking and psychological factors in the human model on the experimental process could be excluded to the greatest extent, which makes the experimental results more reliable. This subject studied the characteristics of age‐related IDD in cynomolgus monkeys, and found that the intervertebral discs of cynomolgus monkeys degenerate with age, which is consistent with the characteristics of human IDD. In addition, this research also found that motor behavior has an interactional relationship with IDD. Motor behavior could be used to infer the presence or extent of IDD in animal models. Therefore, in the future research of IDD, we could confirm the degree of IDD through MRI or Motor behavior (without any manual intervention), and conduct various studies on this basis (such as drug discovery or therapeutic screening). At the same time, changes in Motor behavior could also be used to infer whether the degeneration of the intervertebral disc is aggravated or relieved.

There are several limitations in our study. The knee joint and nervous system may also experience spontaneous degeneration. The influence of these parts on motor behavior could not be ruled out. In future studies, appropriate interventions on IDD will be implemented on the basis of the above work and further observe the relationship between IDD and motor behavior. Although this study has obtained some meaningful results, the number of animals included in the group is relatively small. In subsequent studies, we will increase the number of animals and verify the conclusions of this research on monkeys and humans.

## 5 CONCLUSIONS

In conclusion, this research found that motor behavior has an interactional relationship with IDD. Motor behavior could be used as one of the diagnostic indicators of IDD. It could also be used to infer the presence or extent of IDD in animal models. Avoiding a sedentary lifestyle and engaging in exercise in daily life could alleviate IDD.

## CONFLICT OF INTEREST

The authors declare no conflict of interest. The funders had no role in the design of the study; in the collection, analyses, or interpretation of data; in the writing of the manuscript, or in the decision to publish the results.

## AUTHOR CONTRIBUTIONS

**Wang Jianmin, Zhu Peixuan, Wang Shijun, Chen Dafu, Gao Manman** and **Zhou Zhiyu**: Conception and design of study. **Wang Jianmin, Zhu Peixuan, Wang Wentao** and **Tang Tao**: Acquisition of data. **Pan Ximin, Yang Jun, Wang Shijun, Li Baoliang** and **Zhu Zhengya**: Analysis and/or interpretation of data. **Wang Jianmin**: Writing‐original draft preparation. **Zhu Peixuan, Pan Ximin, Zhu Zhengya** and **Zhou Zhiyu**: Writing‐review and editing. **Pan Ximin, Yang Jun, Gao Manman** and **Zhou Zhiyu**: Supervision. **Chen Dafu, Gao Manman** and **Zhou Zhiyu**: Project administration. **Zhou Zhiyu**: Funding acquisition.
